# Differentiation and functionality of human bronchial epithelial cells in an air-liquid interface culture are modified by irradiation exposure

**DOI:** 10.3389/fpubh.2025.1706687

**Published:** 2026-01-12

**Authors:** Kim Röder, Sylvie Lerchl, David Eilenstein, Carola Hartel, Insa S. Schroeder, Gerhard Thiel, Michael Scholz, Claudia Fournier

**Affiliations:** 1 Department of Biophysics, GSI Helmholtz Centre for Heavy Ion Research, Darmstadt, Germany; 2 Biology, Technical University Darmstadt, Darmstadt, Germany

**Keywords:** lung epithelium, primary bronchial epithelial cells, irradiation, *α*-Particles, X-rays, basal stem cells, air-liquid-Interface

## Abstract

**Introduction:**

We aimed to investigate the effects of *α*-particle and X-ray irradiation on a human bronchial epithelium model, representing environmentally and medically relevant exposure. Our focus was on non-cancer outcomes, namely mucociliary transport (MCT) and epithelial barrier function, both of which are crucial for cancer risk assessment and therapeutic efficacy.

**Materials and methods:**

Basal stem cells were irradiated and terminally differentiated under air–liquid interface conditions into all epithelial cell types. Clonogenic survival assays were used to determine iso-effective doses. MCT was assessed by video tracking of fluorescent bead transport. Cell differentiation was characterized by qPCR for basal, ciliated, goblet, and club cell markers, and mucus composition was analyzed by ELISA for MUC5AC. Barrier integrity was evaluated by transepithelial electrical resistance (TEER) for ion permeability and FITC-Dextran flux for macromolecular permeability. Motility markers were assessed by unjamming transition (UJT) and epithelial-mesenchymal transition (EMT) by morphology and EMT-specific mRNA expression. Inflammatory mediator release was quantified by qPCR and ELISA.

**Results:**

Irradiation reduced bead transport velocity and directedness, indicating impaired MCT. Differentiation marker expression suggested a shift from ciliated to secretory cells, without a corresponding increase in MUC5AC secretion. Barrier function was differentially affected: ion permeability decreased, whereas macromolecular permeability increased. Morphological changes were partially consistent with UJT, but not EMT. Inflammatory mediator levels remained unchanged.

**Discussion:**

MCT impairment did not correlate consistently with the observed differentiation shift. Radiation-induced transition processes, particularly UJT, may underlie the altered permeability. Non-cancer effects were most pronounced at higher doses, with stronger responses to X-ray exposure than to *α*-particle exposure, whereas lower doses, which were still significantly higher than the radiation exposure of a radon spa therapy, had no significant effect.

## 1 Introduction

The respiratory system of vertebrates is constantly exposed to external damage from inhaled particles, such as dust and other particles, and infection by pathogens. In humans, the airway epithelium functions as a physical barrier, protecting the organism, complemented by additional defense mechanisms, such as the mucociliary transport (MCT). The airway epithelium is also part of the immune system, as the secretory cells can release immunomodulatory proteins, i.e., cytokines and defensins, to combat pathogens or to induce an inflammatory response (1–3). Dysregulation of the immune response and a persisting inflammatory response foster chronic inflammatory lung diseases (4, 5). The specific cellular composition and structure of the airway epithelium allow accomplishing these different functions.

External damage is constantly inflicted on the lung epithelium by radiation, which is part of our natural environment and can also be a side effect when radiation therapy is applied to treat cancer and non-cancer diseases (6). In the study presented here, we set out to investigate the impact of ionizing irradiation on the differentiation, functionality, and inflammation-related response of bronchial epithelial cells. The focus lies on *α*-particle exposure, which comes along with natural radioactivity caused by the decay of radon. Radon, a radioactive noble gas that penetrates from rocks and soil into indoor workplaces and homes, is inhaled via the airway system. Radon, therefore, represents the second leading cause of lung cancer after smoking, requiring in-depth knowledge of the mechanisms that contribute to the development of lung cancer, both at the genetic and cellular levels (7–9).

Despite the risk, radon is used for radon spa therapy to treat chronic inflammatory diseases, such as rheumatoid arthritis and ankylosing spondylitis. Furthermore, diseases of the respiratory tract, like asthma bronchiale and chronic obstructive pulmonary disease (COPD), are treated (10). The relevance of understanding the mechanisms of action of treatments of these diseases results from the high incidence or mortality rates. About one-third of the population in Western countries is affected by rheumatoid arthritis (11), while COPD is the third leading cause of death worldwide, followed by lower respiratory infections and lung cancer, which is in sixth place.

The major part of the dose of radon exposure comes from *α*-particles that are emitted by daughter nuclei during radioactive decay. The biological effectiveness of α-particles is high because they release a large amount of energy over a short penetration range, which results in severe and complex DNA damage that is difficult to repair by the intrinsic cellular mechanisms. Experimentally, the induced effects can be investigated *in vivo* using radon chambers (12–14), whereas for *in vitro* experiments, radon gas chambers are not easy to use for an *α*-source, as the dose that can be deposited approaches very low levels, and cell experiments need higher doses compared to organisms to show a measurable effect (12, 15). Therefore, in our experiments, solid-state Americium (^241^Am) was used as an α-source, and X-ray irradiation served as a comparison.

As the lung epithelium was the biological target that we were interested in, we chose in our study a model system for the human bronchial epithelium, the upper part of the airway system. The bronchial epithelium is a pseudostratified luminal layer, consisting of a basal layer of bronchial stem cells, also called basal cells, which is covered by differentiated cells. Basal cells can self-renew and differentiate into the other cell types of the bronchial epithelium, thus ensuring the maintenance of the tissue or replacing damaged cells (16, 17). During the differentiation process of basal cells, ciliated and secretory cells are formed. These two cell types provide a self-cleaning of the airways, also called mucociliary clearance (MCC). The secretory cells produce mucus, which covers the entire surface of the epithelium. Inhaled particles such as dust and pathogens adhere to the mucus and are then cleared by the cilia of the ciliated cells, which is called MCT (18). The MCT also plays an important role in the clearance of radon daughter nuclides that attach to the lung epithelium. Dysfunction of the MCT enhances the dose applied to the lung epithelium and therefore most likely also the risk for lung cancer (10, 19) and fosters infections and the pathogenesis of chronic inflammatory lung diseases (20).

MCC depends on several factors, like the ciliary beat frequency, mucus rheology (viscosity and flow characteristics), generation of the bronchi, abundance, and length of the cilia, which are influenced by the age and health status of the individual (21–24). In view of the relevance of MCC for the development of cancer and inflammatory processes, we used a pseudostratified model of the bronchial epithelium under airlift-culture conditions to investigate the impact of radiation exposure on the differentiation of lung basal cells into functional cells of the lung epithelium *in vitro*.

## 2 Results

The doses that we have selected to investigate the impact of radiation exposure on lung epithelial cells are 2.0 Gy of X-rays, corresponding to the dose of a single fraction in the conventional fractionated radiotherapy of lung cancer (25). For comparison, we used 0.5 Gy of *α*-particles, which is a dose with the same yield of reduced clonogenic survival of lung epithelial cells (“iso-effective” dose), to compare a comparable fraction of surviving cells for both radiation qualities (Supplementary Figure S1). Furthermore, this dose corresponds roughly to the dose that is deposited when one α-particle traverses the nucleus of a cell. Although the overall dose during radon exposure is much lower for the typical exposure scenarios, because only a minor part of the cells is irradiated, the dose to one targeted cell is relevant to understanding the biological effects. For comparison, we also tested 0.5 Gy of X-rays and a dose of 0.25 Gy of *α*-particles. In this case, the α-particle dose does not fully reflect the increased effectiveness, but has been chosen somewhat higher in order to avoid dosimetric uncertainties at very low doses.

### 2.1 Irradiation of basal cells leads to reduced velocity and directedness of mucociliary transport in the differentiated epithelium

To find out whether irradiation of the basal cells alters the functionality of the differentiating lung epithelium, basal cells were irradiated with α-particles and X-rays before the transfer to the air-liquid interface conditions, corresponding to the onset of differentiation. After irradiation, the basal cells were immediately seeded into transwell inserts to expand and differentiate into a bronchial epithelium.

**Figure 1:**
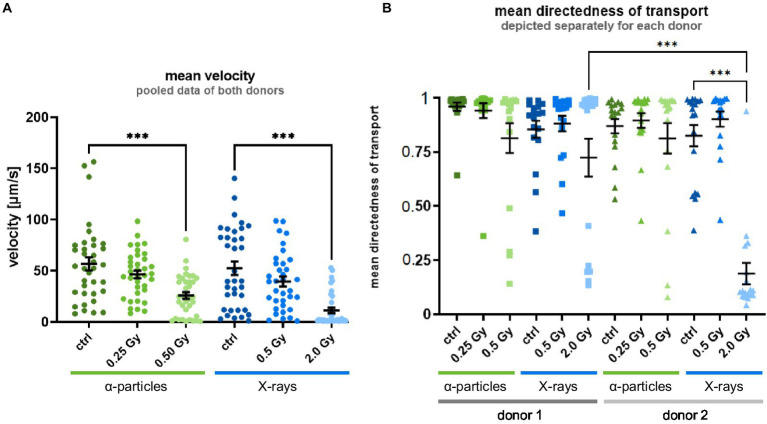
Radiation exposure of the basal cells leads to an impaired MCT at 35 days after the airlift. **(A)** Shows the radiation-induced reduction of the mean velocity of the MCT. The data sets of the donors were pooled because no significant inter-individual difference was observed after irradiation. **(B)** Shows the reduction of the directedness of the MCT. Limiting values of 1 correspond to completely directed movements, i.e., all vectors in a given subregion are exactly parallel, whereas values of 0 correspond to movements equally distributed in all directions in a given subregion. A significant inter-individual difference was observed; therefore, the data sets are depicted separately for each donor. The data are shown as mean ± SEM from three independent experiments per donor, resulting in *N* = 6 for the mean velocity and *N* = 3 for the mean directedness with six individual videos (*N* = 6). Kruskal–Wallis with *post-hoc* Dunn’s test was used for statistical analysis *** *p* ≤ 0.001.

To investigate the hypothesis that irradiation affects MCT in differentiated epithelium, the velocity and directedness of the MCT were measured as parameters for the self-clearance capabilities of the epithelium, which is indispensable to remove inhaled particles from the lungs. Video analysis of the velocity of MCT in controls showed a mean velocity around 55 μm/s for the fully differentiated epithelium (Figure 1A). Irradiation of basal cells with lower doses reduced the MCT velocity slightly for both X-ray and *α*-particle exposure. A reduction of the velocity was significant after exposure to a higher dose for both radiation qualities in comparison to controls. Notably, no significant inter-individual difference was observed after irradiation. Therefore, the data sets of the donors were pooled as presented in the figure.

The second indicator for the functionality of the epithelium that we tested was the directedness of transport. In unirradiated epithelia, the values are close to 1, i.e., transport was almost completely directed (Figure 1B). After irradiation with 0.25 Gy and 0.5 Gy α-particles and 0.5 Gy X-rays, no radiation-induced change was observed compared to the control. Irradiation with a higher X-ray dose resulted in a trend toward or a significant reduction of the directedness of transport for donors 1 and 2, respectively. In contrast to differentiation and velocity of transport, an inter-individual difference for mean directedness in terms of magnitude of reduction was observed after exposure to 2.0 Gy of X-rays in the epithelial cells. Therefore, the data sets are depicted separately for each donor.

Taken together, the radiation-induced reduction of the velocity and the directedness suggests an impairment of the MCT. This could be caused by radiation-induced altered differentiation into functional cells.

### 2.2 Irradiation of basal cells reduces transiently the expression of markers for ciliated cells for high X-ray and *α*-particle doses and enhances the expression of markers for secretory (goblet and club) cells for a high X-ray dose during differentiation into a bronchial epithelium

NHBE were seeded in transwells in a 12-well plate (Corning, 12 mm, 0.4 μm pore, polyester) with 1.1 × 10^5^ cell/cm^2^. After reaching confluence of >80%, the cells were lifted to the ALI by removing the apical media from the transwell, and the basolateral medium was substituted with PneumaCult-ALI medium (STEMCELL) and changed 3 to 4 times per week. Two weeks after the airlift, a mucus wash was performed, and thereafter it was performed every 7 days. Therefore, 500 μL PBS with Ca^2+^ and Mg^2+^ (PBS +/+), prewarmed to 37 °C, was given to the apical side of the epithelium for 5 min. Afterwards, the PBS +/+ is collected for MUC5AC quantification and stored at −80 °C.

Gene expression is known to be affected by ionizing radiation due to DNA damage. The induced lesions can alter various cellular processes, including defense mechanisms against carcinogenesis or differentiation (26).

To reveal radiation-induced alterations in the model used in this study, the expression of cell-type-specific markers was analyzed via RT-qPCR every seventh day throughout differentiation into the typical cell types of the epithelium. The onset of differentiation into differentiated cells of the bronchial epithelium was determined. Furthermore, the cell composition of the developing epithelium was monitored because alterations in cell composition influence the properties of the epithelium and may be a feature of pathological modification *in vivo.*

**Figure 2:**
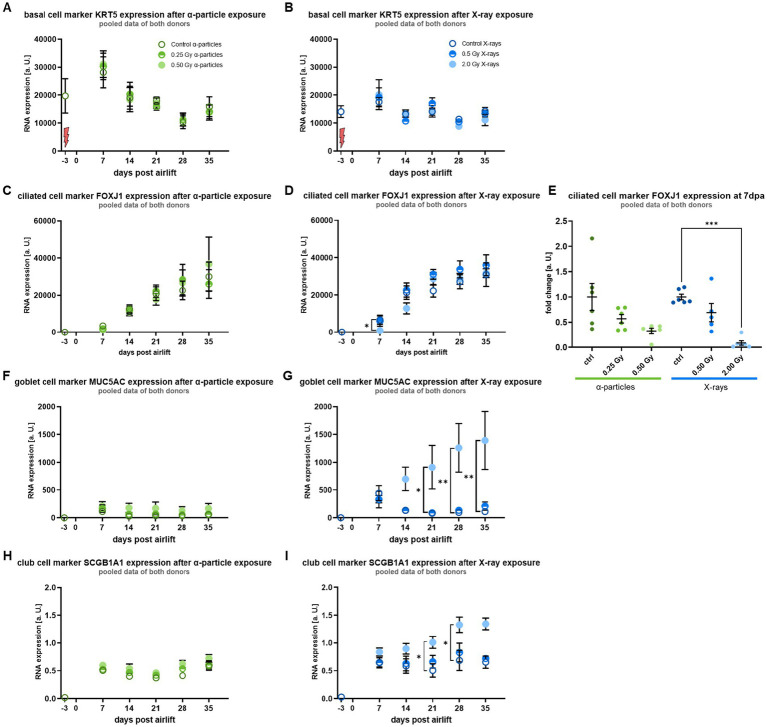
Irradiation leads to a changed differentiation pattern following exposure to a high X-ray dose: Based on gene expression levels during differentiation, ciliated cell markers are reduced, and secretory cell markers are increased after irradiation compared to controls. **(A,B)** Show the unaffected mRNA expression of the basal cell marker KRT5. **(C,D)** Show the mRNA expression of the ciliated cell marker FOXJ1, and **(E)** shows with a higher magnification the results of day 7 post-airlift (dpa), where a reduced expression was observed. **(F,G)** Show the mRNA expression of the goblet cell marker MUC5AC. **(H,I)** Show the mRNA expression of the club cell marker SCGB1A1. Both secretory cell types show, from day 14 on, a higher expression of their respective markers after a high X-ray dose. A red flash marks the time point of radiation exposure (3 days before airlift). Data were normalized to the housekeeping gene RPLPO1 and are depicted as mean ± SEM from three independent experiments per donor, resulting in *N* = 6 with technical triplicates (*n* = 3). Kruskal–Wallis with *post-hoc* Dunn’s test was used for statistical analysis *p ≤ 0.05; **p ≤ 0.01; *** *p* ≤ 0.001.

Figures 2A,B shows that during the differentiation period of five weeks, in unirradiated basal cells, the expression level of the basal cell marker KRT5 was increased at day 7 compared to 3 days before airlift and then decreased over time, indicating the early differentiation of basal cells with stem cell function into secretory cells, such as goblet, club, and ciliated cells (16, 27). Notably, no radiation-induced change was observed.

The expression of FOXJ1, a marker for ciliated cells, increased in unirradiated cells over the differentiation period (Figures 2C,D). After exposure to *α*-particles and X-rays, FOXJ1 showed 7 days after the air lift a lower expression level as compared to controls, which is depicted for day 7 after airlift (Figure 2E). The reduction was less pronounced and only a trend for lower doses (0.25 Gy α-particles and 0.5 Gy X-rays), while it was more pronounced for high doses (0.5 Gy *α*-particles, for 2.0 Gy X-rays significantly reduced). Comparing the expression levels upon irradiation at an isodose of 0.5 Gy, the reduced expression was more pronounced for α-particle and X-ray irradiation, although not significant. From day 14 after airlift, the difference between irradiated and sham irradiated epithelium was not detectable anymore.

In controls, the expression of the marker for goblet cells, MUC5AC, increased rapidly with the onset of differentiation, but decreased to a constant level similar to the initial values (Figures 2F,G), indicating that the differentiation of basal cells into goblet cells starts earlier than the differentiation into ciliated cells. In contrast to the clear radiation response for the expression of FOXJ1 at high doses, the expression levels of MUC5AC showed a radiation-induced increase only for a dose of 2.0 Gy X-rays, and not at all for α-particles. Following irradiation with 2.0 Gy X-rays, the expression level of MUC5AC increased at day 14 post-airlift and was significantly elevated from day 21 onward.

Comparable to MUC5AC, the expression of SCGB1A1, a marker for club cells, increased rapidly in controls at the onset of differentiation and remained constant at this elevated value from day seven after the airlift (Figures 2H,I). The results for both markers of goblet and club cells indicate that in control cells, differentiation into the respective secretory cell types starts earlier than the differentiation into ciliated cells. As for MUC5AC, SCGB1A1 showed a radiation-induced difference in the expression levels only for 2.0 Gy X-rays, where the expression level of SCGB1A1 increased from day 14 on after airlift and was significantly elevated at days 21 and 28 after airlift.

Of note, testing basal cells of two different donors throughout our experiments, no inter-individual difference was observed regarding the expression levels of the different cell markers or the radiation effect. Overall, based on the expression of marker proteins, irradiation reduces transiently the differentiation into ciliated cells, whereas irradiation of basal cells modifies the differentiation into the subtypes of functional cells in a bronchial epithelium, indicating enhanced development of secretory cells.

The regulation of gene expression that influences the differentiation into cell types with different functions is discussed below. As the molecular steps beyond gene expression are even more relevant, we investigated in the next step the release of the mucus protein MUC5AC, to find out more about the impact on the functionality of MCT.

### 2.3 Irradiation of the basal cells did not affect the MUC5AC release into the mucus layer of the differentiated epithelium

The secretion of mucus is known to be a stress response of the bronchial epithelium cells, as the mucus is a protectant, including immune-active molecules (28, 29). However, a persisting hypersecretion of mucus is involved in the pathogenesis of several lung diseases like COPD and cystic fibrosis, as it impairs the MCT (30). As MUC5AC is the major glycoprotein affecting the rheology of the mucus, a hypersecretion of MUC5AC might alter the mucus rheology. In our experiments, the impact of a modified release of MUC5AC on the changes in velocity and directedness cannot be shown unambiguously, because the mucus containing MUC5AC has to be removed for technical reasons before analyzing velocity and directness of the transport. Assuming that the removal of the mucus is not complete and the reduced functionality of MCT after irradiation can still be caused by an altered MUC5AC release by the epithelial cells, we quantified MUC5AC in the fluid resulting from the mucus wash of the epithelia, starting 14 days after the airlift.

**Figure 3:**
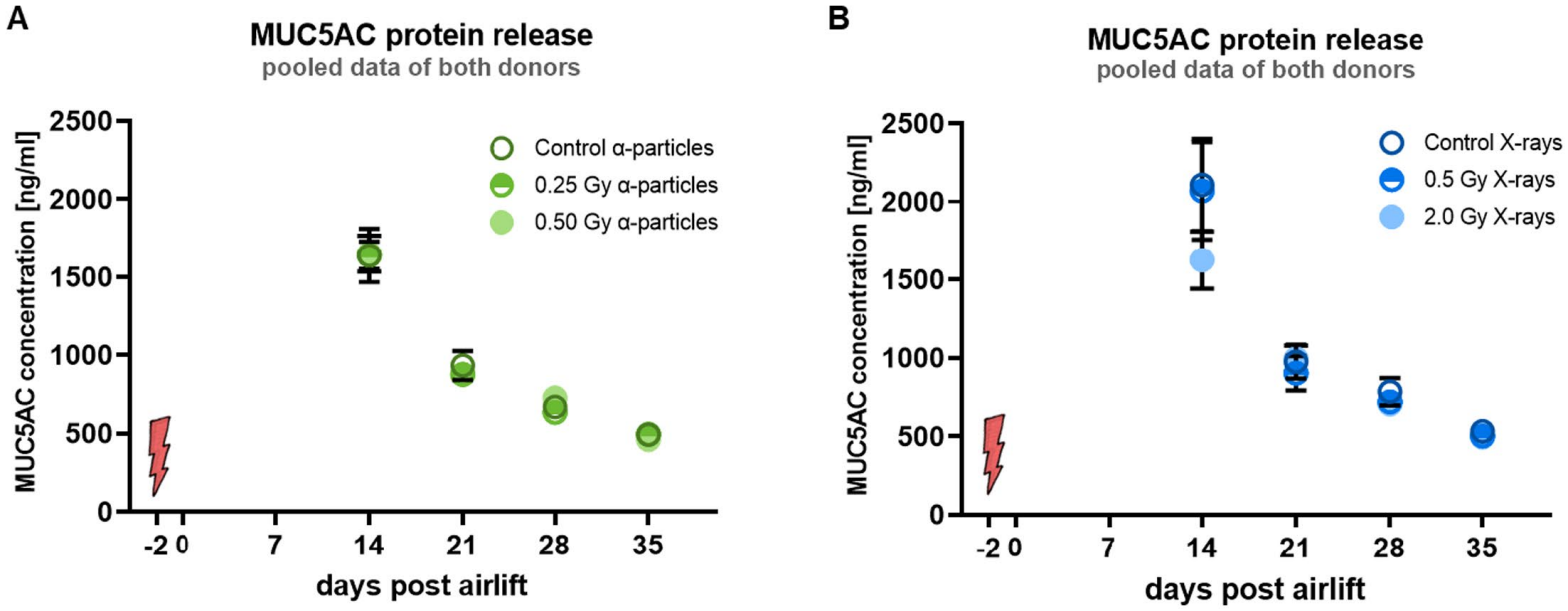
Irradiation of the basal cells did not alter the amount of released MUC5AC protein. **(A,B)** Show the concentration of MUC5AC protein in the liquid from the mucus wash. Red flash marks the time point of irradiation. Data are depicted as mean ± SEM from three independent experiments per donor, resulting in *N* = 6 with technical triplicates (*n* = 3). Kruskal–Wallis with *post-hoc* Dunn’s test was used for statistical analysis.

In controls, the release of MUC5AC was maximum at 14 days after airlift and decreased over time during the differentiation period (Figures 3A,B). Notably, no radiation-induced change was observed. Since irradiation did not lead to any change in MUC5AC release, this suggests that an altered MUC5AC concentration in the mucus is not a reason for the observed reduced functionality of the MCT. Remarkably, the reduced MUC5AC release over the differentiation period does not correspond to the increased mRNA expression level of MUC5AC, suggesting that translational or posttranslational modifications of the MUC5AC protein production do not follow the changed gene expression after higher doses, i.e., 2.0 Gy of X-rays. Taken together, our results suggest that changes in the mucus release are most likely not a reason for the impaired functionality after irradiation, but a causal relationship between mucus production and MCT movement is still possible, as we measured the movement for technical reasons only after washing away the mucus.

In a next step, we investigated additional markers of the functionality of the epithelium, i.e., the barrier function of epithelium after irradiation.

### 2.4 Radiation modifies the barrier function of the epithelium after exposure of basal cells to higher doses of *α*-particles and X-rays

The barrier function of the lung epithelium is one of its key functions to prevent pathogens from entering the lung tissue (2), but also to control the transport of molecules. Based on the results shown above, α-particle and X-ray exposure reduce the MCT of epithelia, increasing the retention time of pathogens or airborne particles like radon decay products on the epithelium and providing more time to penetrate the epithelium. Thus, the integrity of the epithelium is necessary to protect the tissue from the entry of pathogens and molecules.

To analyze the barrier integrity of the epithelium, the permeability of the epithelium was assessed. Epithelial permeability is determined by two processes: transcellular transport through the cell, from the apical to the basolateral membrane, and paracellular transport through the intercellular space between the cells. The paracellular transport is a diffusion process along a transepithelial electrochemical gradient and is controlled by tight junctions (31). This transport can be assessed by measuring the transepithelial flux of ions and of macromolecules.

In our study, the paracellular permeability of the fully differentiated epithelium was quantified 35 days after airlift by measuring the transepithelial electrical resistance (TEER), which reflects ion flux across the epithelial layer. Furthermore, the flux of the fluorescence-marked tracer molecule FITC dextran, indicating the transport of macromolecules, was measured. An increased TEER and a decreased FITC dextran flux would indicate a reduced paracellular permeability (31).

**Figure 4:**
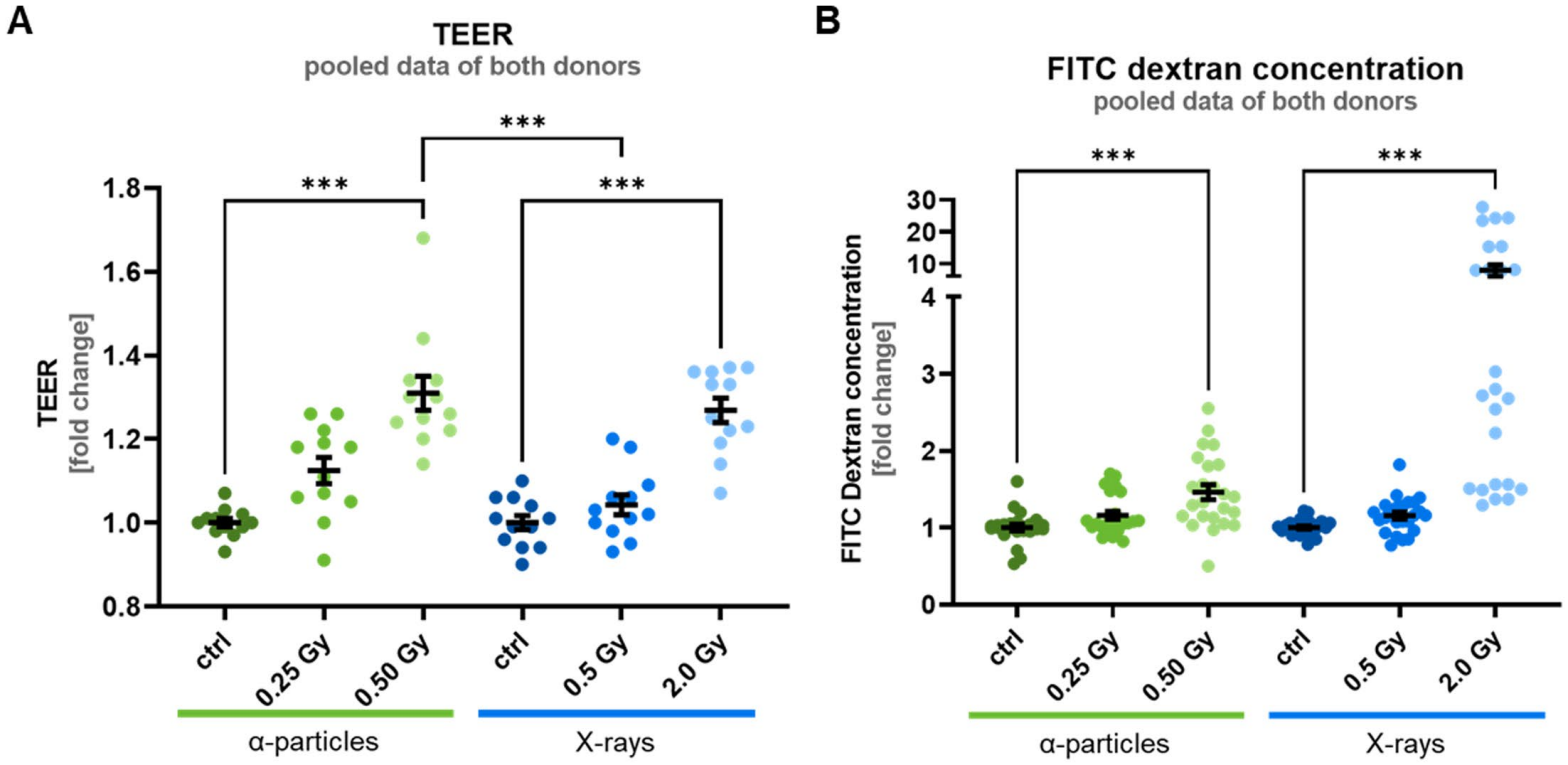
Irradiation of the basal cells leads to an increased TEER—indicating a reduced ion conductivity- and an increased FITC dextran flux - indicating an increased permeability for macromolecules at 35 days after the airlift. **(A)** Shows the increased TEER with a significant difference between X-ray and α-particles, at an isodose of 0.5 Gy. **(B)** Shows increased FITC dextran permeability after irradiation. Data are depicted as mean ± SEM from three independent experiments per donor, resulting in *N* = 6 with two analyzed epithelia (*n* = *n*). Kruskal–Wallis with *post-hoc* Dunn’s test was used for statistical analysis ***p ≤ 0.001.

Measurements of TEER on fully differentiated epithelia from two donors revealed resistance values of 180 and 410 *Ω* cm^2^. These values are typical for TEER measurements of epithelial layers (32, 33), although somewhat lower than those reported previously for fully differentiated airway epithelia (34) (ref.). After exposure to *α*-particles and X-rays, cells revealed a dose-dependent increase in TEER compared to controls, which is significant after exposure to high doses of both α-particles and X-rays (Figure 4A). This change corresponds to a reduced transport of ions and a reduced permeability of the epithelial layer. An increase in the electrical resistance of the epithelial layer suggests that the respective doses of radiation are neither causing apoptosis nor necrosis at the time of the investigation. Notably, both forms of cell death are known to reduce the electrical resistance of epithelial layers (35). Comparing the TEER for an isodose of 0.5 Gy, α-particles lead to a significantly higher elevation of TEER compared to X-rays; thus, α-particles are more efficient in reducing the permeability. Regarding inter-individual differences, the TEER of controls differs for the donors in the absolute values, i.e., donor 2 shows a more than twofold higher TEER value. However, the relative change in TEER in epithelia of irradiated cells compared to control did not differ for the donors, resulting in similar radiation effects.

Complementary measurement of paracellular flux of FITC dextran recorded at the same time point as the TEER data (35 days after airlift) shows no significant radiation-induced changes for lower doses (Figure 4B). Surprisingly, while the TEER data predict a higher resistance of the epithelial layer after irradiation, we observed a significant elevation of transepithelial FITC-Dextran diffusion for the high X-ray dose tested. While the data exhibited an inter-individual difference, i.e., a 50% higher value for donor 2 compared to donor 1, the relative increase in FITC-dextran permeability of epithelia from irradiated cells compared to control was similar.

As explained in the next paragraph, the observed alterations in permeability of the epithelial layer could be related to altered cell motility. In the next step, we investigated the cellular mechanisms leading to a higher cell motility, i.e., the “unjammed transition” (UJT) reported to occur after irradiation exposure (36) and the epithelial-mesenchymal transition (EMT), known to occur after irradiation in the presence of the cytokine TGF-*β* (37).

### 2.5 Irradiation of the basal cells can lead to morphological changes related to changes in motility in the differentiated epithelium

The cells of an epithelial layer are organized in a fixed network, i.e., they do not move relative to each other; they are in a “jammed” state. Under certain conditions, epithelial cells can become mobile or “unjammed” to ease collective and cooperative cell migration, while partially maintaining the junctional integrity. The transition between the two states, the so-called “unjammed transition” (UJT), has been described for different stages of cell development, wound healing, metastasis formation of epithelial tumors, and, recently, also as part of the response to irradiation. This transition is related to changes in the permeability of the epithelial cell membranes (36, 38).

Similar to EMT, UJT is characterized by an altered morphology, i.e., an elongation of cell shape (36, 38, 39). Therefore, in the next step, the cell shape of the epithelial cells was analyzed.

If UJT is induced, epithelial cells show an elongated cell shape. To assess this after irradiation, the cell shape of the epithelial cells was examined for elongation. An elongation of the cells is characterized by modified morphological parameters, i.e., an increased aspect ratio and shape index. To distinguish between UJT and EMT as a trigger for the elongation, the E-cadherin concentration in the cell membrane was analyzed, as EMT is characterized by a reduction of E-cadherin expression (38).

**Figure 5:**
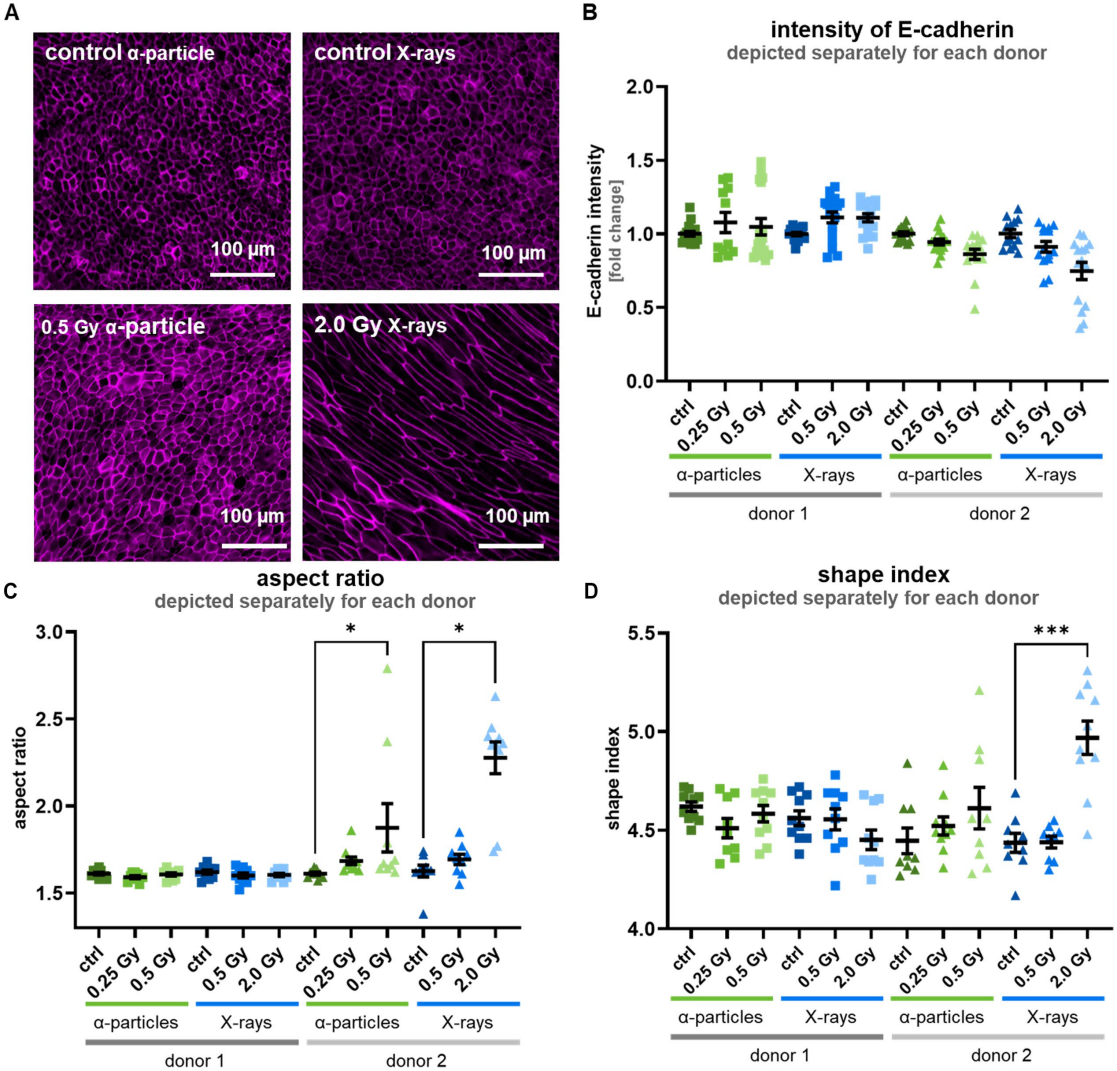
Irradiation of the basal cells can lead to morphological changes of the cells 35 days after airlift. **(A)** Shows for donor 2 the immunofluorescent staining of the tight junction protein occludin; the cell shape of control cells is rounded, while it is almost unchanged after exposure to a higher dose of α-particles, and clearly elliptical and elongated after exposure to a high X-ray dose, consistent with the radiation effect observed after analysis of aspect ratio and the shape index shown in **(C,D)**. **(B)** Shows the measured intensity of the adherens junction protein E-cadherin, which was not altered after radiation exposure. **(C)** Shows the aspect ratio of the cells where no radiation effect was visible in cells of donor 1, but for donor 2, an increase was observed at higher doses. **(D)** Shows the shape index of the cells, where no change after irradiation was observed in cells of donor 1, but for donor 2, at a higher X-ray dose, an increase was measured. Data are depicted as mean ± SEM from three independent experiments separated after donor, resulting in *N* = 3 with five analyzed images (*n* = typically 5). Kruskal–Wallis with *post-hoc* Dunn’s test was used for statistical analysis *p ≤ 0.05; ***p ≤ 0.001.

To visualize the cell morphology, the epithelia were stained at 35 days post-airlift for the tight junction protein occludin (Figure 5A, shown for donor 2) and the adhesion junction molecule E-cadherin. For both donors, the fluorescence intensities of the adhesion junction molecule E-Cadherin in the progeny of unirradiated as well as *α*-particle and X-ray exposed cells did not show significant changes in the fluorescence intensity of E-cadherin (Figure 5B).

For the cell shape analysis, the pictures of the occludin staining were used. Morphological parameters such as perimeter, area, and minor and major axes of each cell were measured by dedicated software (see materials and methods). The measured parameters were used to calculate the aspect ratio, calculated as the ratio of major to minor axis, and the shape index, calculated as the ratio of perimeter to area. As depicted in Figures 5C,D, for donor 1, the analysis of the aspect ratio and shape index revealed no radiation-induced alterations, while for donor 2, increases in the aspect ratio and the shape index after exposure to a high X-ray dose and after exposure to a higher *α*-particle dose were detected at least for the aspect ratio, but not for lower doses of X-ray and α-particles. This shows clear inter-individual differences, i.e., for one out of two donors, a higher proportion of elongated cells in the epithelium after exposure to high doses.

Taken together, our results on morphological changes are consistent with a radiation-induced UJT, while the lack of changes in the E-cadherin expression does not indicate EMT.

## 3 Discussion

To contribute to an improved understanding of the biological mechanisms underlying the risk of environmental exposure, the goal of this study was to investigate the impact of two radiation qualities, i.e., α-particles and X-rays, on the capacities of bronchial stem cells of the basal layer to differentiate into a functional epithelium. In our irradiation experiments, an isosurvival dose was used to compare the effects of both radiation qualities, i.e., 0.5 Gy α-particles and 2.0 Gy X-rays, as well as a lower dose of 0.25 Gy α-particles and 0.5 Gy X-rays.

Specifically, we aimed to examine the capacity of bronchial stem cells after irradiation to perform an efficient MCT to remove inhaled particles from the epithelial surface, and the maintenance of the barrier function, i.e., the permeability of the epithelium. To address this, we used as a model for bronchial epithelium *in vitro* cultures of bronchial, basal stem cells, isolated from the human sternum. In this model, the stem cells differentiated under air-liquid-interface (ALI) culture conditions into a functional and organotypic bronchial epithelium. Amongst others, Boei et al. have described the good suitability of a lung ALI culture, including club, ciliated, and goblet cells, for the investigation of toxic effects of airborne substances. Interestingly, the authors also report inter-individual differences in the results obtained for cells of different donors (40).

With respect to the functionality of MCT and in contrast to lower doses, the higher doses of both radiation qualities tested showed a reduction in the velocity and directedness of MCT compared to controls. The deteriorated MCT suggests a radiation-induced impairment of mucociliary clearance *in vitro*. Additional evidence for this comes from side effects observed for high doses of X-rays (18–54 Gy) administered in the course of radiotherapy of head and neck cancer in children: Surico et al. showed a long-term reduction of the nasal MCT and increased occurrence of airway infections (41, 42). As a consequence of a dysfunctional MCT, it has been shown *in vivo* that longer retention time of pathogens and radon decay products in the lung by an impaired MCT fosters the pathogenesis of chronic inflammatory diseases (43, 44). Conversely, for very low activity concentrations, corresponding to very low doses, Passali et al. showed a more efficient nasal MCT in patients with perennial allergic rhinitis and chronic sinonasal inflammation after 14 days of inhalation or use of a nasal spray containing radon-enriched thermal water. The authors discuss the beneficial effect of low doses of alpha particle exposure for chronically ill patients (45–47).

In the next steps, we intend to find reasons for the impairment of MCT function. We first hypothesized that irradiation of basal cells leads to an altered differentiation pattern that, in turn, impacts the functionality of the epithelium. At this point, it should be mentioned that with respect to the differentiation process, the reliability of the model was excellent, because even after exposure to the higher doses tested, i.e., 0.5 Gy *α*-particles and 2.0 Gy X-rays, basal cells were able to differentiate into an epithelium with normal morphology, but altered functionality. In contrast to the lower X-ray and both α-particle doses tested, we could show for the higher dose of X-rays (2.0 Gy) a modified pattern of differentiation into the subtypes of the functional cells, i.e., a transiently lower expression of markers for ciliated cells of the bronchial epithelium, pointing to a delayed ciliogenesis. For both X-rays and *α*-particle irradiation, a radiation-induced increase in marker expression of cellular key players of the MCT, i.e., secretory cells (goblet and club cells), was observed, indicating hyperplasia of these cells.

These results are consistent with previously published results on X-ray irradiation of bronchial stem cells with doses exceeding the dose range tested in our work. Here, also an altered expression of ciliated and secretory cell type markers occurred in the differentiating epithelium, discussed as a NOTCH-mediated effect (48, 49). Notch signaling is a key pathway for the inhibition or stimulation of epithelial cell differentiation at different stages of lung development and, amongst others, maintains a balance between the differentiation of secretory and ciliated cells. Upon severe damage, the epithelium is regenerated via proliferation of progenitor cells or by dedifferentiation of adult epithelial cells. This is not the case for ciliated cells (50), which could explain the decrease in marker protein for ciliated cells after irradiation observed in our study.

Interestingly, the radiation response related to the expression of differentiation markers shows a different pattern than that of clonogenic survival. In contrast to the markers for ciliated cells, the markers for mucus-producing, secretory goblet and club cells increase with X-ray dose. It can be assumed that with increasing damage induced by 2 Gy X-rays compared to 0.5 Gy, the cells tend to differentiate into secretory cells to activate the MCT. This is not the case for *α*-particles; thus, it is in line with other observations that the increased effectiveness of α-particles with respect to cell killing is not necessarily reflected in other endpoints (51).

Concerning the possible impairment of functionality, a dysregulation of differentiation due to radiation may lead *in vivo* to an impaired regeneration process of the tissue after an injury, as it reduces the differentiation capabilities of the stem cells and increases senescence (6, 48, 52, 53). As a consequence of the shift of cellular markers in the differentiating epithelium, we hypothesized that one reason for the radiation-induced impaired functionality of MCT could be the content of glycoproteins in the mucus, namely MUC5AC. This glycoprotein is synthesized by goblet cells and is a key component of the MCT (54, 55). The information about the expression of MUC5AC is missing in our study, because due to a limited cell number in the ALI culture, the protein expression has not been investigated. However, despite a transiently increased expression of the MUC5AC expression marker, no changes in the protein release have been detected. This argues against an impact of the increased abundance of secretory activity by the respective cell types on the observed impaired MCT functionality. As a result of this part of our study, the mechanistic basis of the observed impairment of MCT function remains to be fully clarified. However, the abundance of the ciliated cells has not been assessed when the functionality is impaired, which could be the mechanistic basis of the reduced velocity and directness of the transport.

With respect to the barrier function of the bronchial epithelium, our measurements revealed no significant changes at low doses, while higher doses of X-rays and α-particles resulted in reduced permeability for ions (increased TEER), but an increased transport of macromolecules (increased FITC dextran flux) from the apical side of the differentiated epithelium across the epithelium itself. While these data confirm our general hypothesis of a radiation-induced change of epithelial functionality, they provide at this point no clear-cut explanation for the processes underlying transepithelial transport modifications. If both ion and macromolecule transport would be affected in a similar manner, an increased TEER should occur concomitant with a decreased FITC dextran flux, e.g., in the case of a reduced paracellular permeability and vice versa (31). However, in our case, the data indicate changes to be opposed for ions versus macromolecules for both radiation dependence and interindividual differences. Experimental evidence has been provided for different courses of FITC-Dextran and resistance measurements over time, endorsing our experimental data (56).

A speculative explanation for this conundrum could be that high doses of radiation favor the permeation of large molecules through the paracellular route. A slow diffusion of FITC would at the same time block the ion current through this pathway and increase in this manner the electrical resistance. Albeit hypothetical such a scenario is possible and can be observed when ions compete with either proteins or DNA for a passage through narrow pores (57, 69). An alternative explanation of the conflicting electrical and dextran diffusion data could be that irradiation affects multiple transepithelial transport pathways, increasing the transepithelial resistance by strengthening the tight junctions. At the same time, irradiation could stimulate a vesicle-mediated transcellular transport, i.e., transcytosis, which occurs in epithelial cells in particular under stress (58, 59); this could increase the transfer of large molecules across the cell layer.

To obtain more insight into the consequences of the observed increased permeability of the epithelial layer, at least for macromolecules, we investigated the UJT transition process that is related (36, 38, 39). UJT is associated with a typical elongated cell morphology, which we found in only one of two donors. As reduced permeability was found in both donors, the occurrence of UJT is not fully supported by our data. However, UJT induction was reported after repeated X-ray exposure testing using a completely different experimental design (36).

Moreover, the consequences of a reduced permeability of the epithelium, as shown for ions in our study, are not entirely clear. It is likely that inflammation occurs: Wawrzyniak et al. showed a correlation between altered permeability and asthma and concluded that reduced barrier function fosters asthma pathogenesis (60), which Coyne et al. showed for cystic fibrosis (61). Modified barrier function can influence the infection risk, pathogenesis of chronic inflammatory diseases, and penetration potential for immune or cancer cells (2). Therefore, because the functionality of the physical barrier of the epithelium is also affected by cytokines and growth factors, which modulate the immune response of the epithelium (62), we investigated as a next step the impact of irradiation on the release of inflammatory cytokines. However, no significant radiation-induced changes related to inflammation or a senescence-associated secretory phenotype were observed (Supplementary, Figure S2).

This might be due to the lack of resident or attracted immune cells in the model system of the lung epithelium that we used in our study, not reflecting the dynamics between immune and epithelial cells that is established when the epithelium is damaged. This is in contrast to the model system of human skin, where inflammation was observed even in the absence of immune cells (63). Another explanation for the absence of inflammation-related changes in our study is that we tested relatively low doses. This idea is endorsed by studies from other groups: Chauhan et al. showed in the lung cancer cell line A549 that after 1.5 Gy *α*-particle, altered expression of several proinflammatory cytokines, while after 1.5 Gy X-rays, only IFNγ was downregulated (64). In a different context, i.e., the influence of nicotine on the α-particle induced radiation response in bronchial epithelial BEAS-2B cells, Boroumand and co-workers reported increased IL-6 and IL-1β mRNA levels post-treatment (65). With bronchoalveolar lavage of lung cancer patients, Barthelemy-Brichant et al. showed after a total dose of 20–60 Gy in 2-Gy fractions of X-rays an increase of IL6 for over six months and concluded that IL6 is involved in the radiation response of human lung tissue (66). Thus, a large body of data indicates the involvement of inflammation in the radiation response of the lung. However, the results of our study obtained for relatively low doses, i.e., unchanged release of cytokines and MUC5AC production, are consistent. We conclude that cytokine release is unlikely to contribute to the observed altered paracellular permeability after the irradiation doses tested in our study.

The question arises whether our observations of MCT dysfunction, reduced MUC5AC expression, and an increase in TEER could be a consequence of cytotoxicity of X-ray and *α*-particle exposure. However, this can be excluded because the cell growth of the surviving cells increases with increasing time gap between irradiation and analysis; daughter cells overgrow the directly irradiated cells. Also, an increase in the electrical resistance of the epithelial layer suggests indirectly that the respective doses of radiation are neither causing apoptosis nor necrosis over the time of the investigation. Notably, both forms of cell death are known to reduce the electrical resistance of epithelial layers (35). Furthermore, increased expression of markers of secretory cells was observed only after X-ray irradiation, whereas *α*-particle exposure did not modify the expression of markers. This renders the explanation based on cytotoxicity very unlikely, since doses were chosen to be iso-effective in cell killing.

Taking all our results together, we showed that irradiated basal cells are able to form a pseudostratified epithelium, which, however, was altered in its function With respect to the MCT, a clear impairment after the higher doses, i.e., 0.5 Gy α-particles and 2.0 Gy of X-rays, was observed. Also, the permeability of the epithelium showed a radiation-induced change after irradiation with higher doses; the impact on the barrier properties of the epithelium has to be further investigated. The described radiation-induced effects were observed only after the higher doses, similar to typical doses used in one fraction of radiotherapy. Remarkably, the lower doses (0.25 Gy α-particles, 0.5 Gy of X-rays), which are significantly higher than the radiation exposure of a radon spa therapy (10, 19), had no significant effect on the functionality of the epithelium.

## 4 Materials and methods

### 4.1 Submerged cell culture

Normal primary human bronchial epithelial cells (NHBEs) from two different donors (Lonza; Wokingham, UK) were cultured in PneumaCult Ex Plus medium (STEMCELL Technologies Inc., Vancouver, BC, Canada). Basal cells were characterized by immunofluorescence staining of the basal cell marker KRT5. Media change was performed 3 to 4 times per week. Irradiation was performed in passage 3 with an approximate confluence of 80%.

### 4.2 Irradiation of primary human bronchial epithelial cells

At the time of X-ray irradiation, the basal cells were maintained in a T25. X-ray exposure was performed at room temperature at an MXR 320/26 X-ray tube (Comet Holding AG, Wünnewil-Flamatt, Switzerland; 250 kV and 16 mA, 2.7 Gy/min). A filter set of 3 mm Be, 1 mm Al, and 1 mm Cu was used to harden the energy spectrum.

Controls were sham-irradiated. Passage into the 12-well transwell plates (Corning, 12 mm, 0.4 μm pore, polyester) was performed within 1 h after the exposure.

For α-irradiation, the basal cells were grown on a 4 μm thin biaxially-oriented polyethylene terephthalate (boPET) foil treated with air plasma under atmospheric conditions. The α-irradiation was performed by using an americium (^241^Am) source (α-particles with an energy of 5.486 MeV) in a setup described by (15). For dosimetry, in a first step the alpha particle fluence was determined by means of nuclear track detectors which were positioned at the same distance from the source as the cell samples. The corresponding energy spectrum was measured using a surface barrier solid-state detector at the same position. From the energy distribution, the LET distribution could then be determined using the energy loss program ATIMA. Combining the fluence and LET information yields a dose rate of 8.2 ± 2.4 Gy/min.

Controls were also sham-irradiated and plated into the 12-well transwell plates (Corning) within 1 h of exposure.

### 4.3 ALI culture

NHBE were seeded in transwells in a 12-well plate (Corning, 12 mm, 0.4 μm pore, polyester) with 1.1 × 10^5^ cell/cm^2^. After reaching confluence of >80%, the cells were lifted to the ALI by removing the apical media from the transwell, and the basolateral medium was substituted with PneumaCult-ALI medium (STEMCELL) and changed 3 to 4 times per week. Two weeks after the airlift, a mucus wash was performed, and thereafter it was performed every 7 days. Therefore, 500 μL PBS with Ca^2+^ and Mg^2+^ (PBS +/+), prewarmed to 37 °C, was given to the apical side of the epithelium for 5 min. Afterwards, the PBS +/+ is collected for MUC5AC quantification and stored at −80 °C.

### 4.4 RNA isolation and quantitative reverse-transcriptase PCR

Residual cells from seeding into transwell plates were washed with PBS and dissolved in QIAzol® lysis reagent (QIAGEN, Venlo, Netherlands). After the airlift, every 7 days epithelia from two inserts were collected for RNA isolation by detaching the cells in PBS and dissolving the cells in QIAzol® lysis reagent (QIAGEN). Samples were stored at −80 °C till isolation. RNA isolation was performed with the RNeasy Mini Kit (QIAGEN) according to the user’s manual, including a DNA digestion with the RNase-free DNase set (QIAGEN). The mRNA was transcribed into cDNA with the RevertAid RT Reverse Transcription kit (Thermo Scientific, Waltham, MA) according to the user’s manual. Real-time qRT-PCR was performed in a Quant Studio 3 (Thermo Scientific) using the following primers, which were synthesized by Biomers (Ulm, Germany): RPLPO1 forward 5′-CCTCGTGGAAGTGACATCGT-3′; RPLPO1 reverse 5′-CTGTCTTCCCTGGGCATCAC-3′; KRT5 forward 5′-AGGAATGCAGACTCAGTGGAGAAG-3′; KRT5 reverse 5′-; TGCCATATCCAGAGGAAACACT-3′; FOXJ1 forward 5′-ACTCGTATGCCACGCTCATCTG-3′; FOXJ1 reverse 5′-GAGACAGGTTGTGGCGGATTGA-3′; MUC5AC forward 5′-TGTGAGGGCAACAACGTCAT-3′; MUC5AC reverse 5′-CCATCTTGGTCAGCCACCTT-3′; SCGB1A1 forward 5′-AAAGCATCATTAAGCTCATGGAAA-3′; SCGB1A1 reverse 5′-TGGAGCAGTTGGGGATCTTC-3′; IL1β forward 5′-CCACAGACCTTCCAGGAGAATG-3′; IL1β reverse 5′-GTGCAGTTCAGTGATCGTACAGG-3′; IL8 forward 5′-GCGCCAACACAGAAATTATTGTAA-3′; IL8 reverse 5′-TTCTTGGATACCACAGAGAATGAA-3′.

### 4.5 Protein quantification

Protein quantification of the glycoprotein MUC5AC in the PBS +/+ from the mucus wash was performed using a human MUC5AC ELISA Kit (NBP2-76703, Bio-Techne, Minneapolis, MN) according to the manufacturer’s instructions. The cytokines IFNγ, IL1β, IL6, MCP1, and TNFα were quantified by a U-Plex® Custom Multiplex Assay (Meso Scale Discovery, Rockville, MD) of the culture medium according to the manufacturer’s instructions.

Please note that the number of viable cells contributing to protein release was not determined because the release was measured over time. At each time point, mucus was taken to run the different ELISA tests, and the ALI culture was continued. This represents an unavoidable limitation of the method.

### 4.6 Immunofluorescent staining

For immunofluorescent staining, epithelia were washed with PBS −/− and fixed for 15 min in 3.7% paraformaldehyde. The epithelia were washed again with PBS −/− and permeabilized for 20 min in 0.5% TritonX and 1% BSA. Blocking was performed for 90 min in 1% BSA, 10% goat serum, 1% Tween 20, and 0.3 M glycine. Staining was performed with 1:30 E-cadherin (DECMA-1, Alexa Fluor™ 488, Invitrogen of Thermo Scientific, Waltham, MA) and 1:75 Occludin (OC-3F10, Alexa Fluor™ 594, Invitrogen) in the blocking solution for 90 min in the dark at room temperature. Epithelia were washed three times for 5 min with PBS −/−. Nucleus staining was performed with 1.5 mg/mL DAPI for 15 min in the dark, and afterwards the epithelia were washed with PBS −/−. Epithelia were mounted with Dako Mounting Medium (Agilent Technologies, Santa Clara, CA). Fluorescence images were taken at the fluorescence microscope Axio Imager. Z2 (Carl Zeiss AG, Oberkochen, Germany) with the CCD Kamera CoolCube4 (MetaSystems, Heidelberg, Germany).

### 4.7 Cell shape analysis

Analysis of cell morphology was performed using the Cell Image Analyzer (Yannic Röder Engineering, Darmstadt, Germany). Background-corrected images of the occludin staining were converted into binary images, background noise was removed, and the contours were additionally sharpened. The contours of the individual cells were determined with a watershed-based algorithm, and an ellipse was fitted onto each cell. Subsequently, the various parameters of the individual ellipses of the cells were calculated as described previously by Park *et al.* (67). Including the aspect ratio, which was calculated by the division of the major axis by the minor axis (Formula 1), and the shape index, where the perimeter of the ellipse was divided by the square root of the area (Formula 2).


$$\;\text{aspect ratio}=\frac{\text{major axis}}{\text{minor axis}}$$ (1)

$$\;\text{shape index}=\frac{\text{perimeter}}{\sqrt{\text{area}}}$$ (2)

The mean aspect ratio and shape index were calculated from all complete cells within the image and amount of normal and elongated cells is given.

### 4.8 TEER

**Figure 6:**
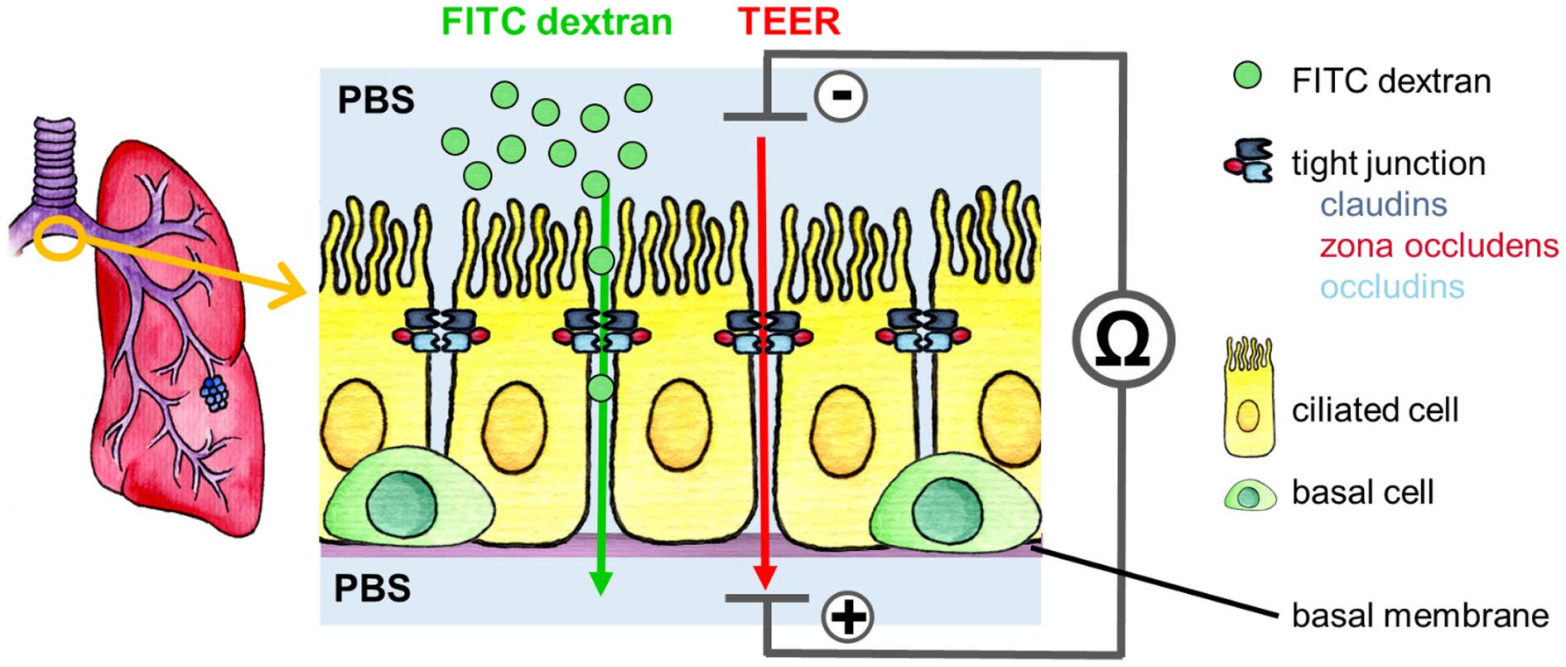
Paracellular permeability measurement via FITC dextran flux assay (green arrow) and TEER (red arrow) of the bronchial epithelium and its organotypic orientation. To measure TEER, a current is applied to the epithelium, which is in an isotonic electrolyte solution (PBS). The resistance of the epithelium to the current is measured. It is a parameter of the paracellular ionic conductivity through the epithelium, which is mainly regulated by the tight junctions. For the FITC dextran permeability assay the epithelium is also in PBS, but at the apical side FITC dextran is added. During the incubation, the FITC dextran macromolecules are actively transported through the paracellular space, which is regulated mainly by tight junctions.

The assessment of paracellular permeability is explained in Figure 6.

TEER was measured according to the manufacturer’s instructions in ALI cultures at 35 dpa using an Epithelial Tissue Voltohmmeter (EVOM) with EndOhm-12 circular disk electrodes (World Precision Instruments, Sarasota, FL). Briefly, using standard Dulbecco’s modified PBS without Ca^2+^ and Mg^2+^ prewarmed to room temperature as an electrolyte. After a 10-min equilibrium, the TEER value was measured. TEER is expressed as *Ω* × cm^2^.

The mucus wash was suspended 1 week before analysis to keep the integrity of the cellular layer, and the measurement was carried out in the presence of the mucus.

### 4.9 FITC dextran flux assay

Paracellular permeability was assessed by a 4 kDa FITC dextran assay (Sigma-Aldrich of Merck Group, Darmstadt, Germany) following the reference (68). With the subsequent modifications at 35 dpa, 500 μL PBS −/− were added to the well basal to the transwell insert, and 200 μL of 4kD FITC dextran at a concentration of 5 mg/mL was added apical to the epithelium. After a 30-min incubation at 37 °C, 5% CO_2_, and 95% relative humidity, a fluorimeter (SpectraMax MiniMax 300 Imaging Cytometer, Molecular Devices, San Jose, CA) was used to determine the fluorescence of 100 μL PBS taken from the well. Concentration of FITC dextran was calculated from the standard curve using the 4-parameter fit of GraphPad Prism 9 software (GraphPad Software Inc., San Diego, CA).

For the TEER measurement, the mucus wash was suspended 1 week before analysis to keep the integrity of the cellular layer, and the measurement was carried out in the presence of the mucus. Equally relevant to the TEER measurement, the cell occupancy rate of the transwell membrane has been visually assessed at the time points of analysis and is estimated to be 100%.

### 4.10 Video-based analysis/measurement of transport properties

Videos of the apical side of the fully differentiated epithelium were taken 35 days post-airlift (dpa) at room temperature. Mucus from the epithelia was removed shortly before measurement by a 5-min wash with 5 mM DTT in PBS, followed by 5-min washing steps with PBS. Green fluorescent beads with a diameter of 3 μm (Sysmex Deutschland GmbH, Germany, Concentrate Cod. 05–4,008, 488 nm excitation) were diluted in PBS, and 15 μL of the dilution was put on top of the washed epithelium. After an incubation time of 15 min at 37 °C for each epithelium, at three randomly selected sites, a two-minute video (44 frames per second, object field of 1,350 × 1,083 μm; 1.46 mm^2^) was recorded with the Echo Revolve (ECHO a bico company, San Diego, CA) using the FITC LED light cube and the 5 × objectiv LMPlanFLN (Olympus, Tokyo, Japan).

Video-based analysis of the transport of the beads was performed using the in-house developed software ImageD^1^ and the cilia microsphere tracker plug-in. The software localizes the single beads and determines their movement, characterized by a shift vector, if they are present in at least 12 successive frames. The length of the shift vector describes the velocity v, and the angle of the direction of transport.

Directionality of movements is determined from the weighted mean of normalized direction vectors in multiple subregions, where small-scale movements can be considered linear in a good approximation. We therefore performed the analysis separately in 10 × 8 subregions of the field of view to avoid distortions resulting from the regular large-scale movement patterns. The weighted average of directionality is calculated by the mean of the 80 subregions weighted by the number of movements observed in each subregion. Limiting values of 1 correspond to completely directed movements, i.e., all vectors in a given subregion are exactly parallel, whereas values of 0 correspond to movements equally distributed in all directions in a given subregion.

### 4.11 Statistics

Statistical analysis was performed in GraphPad Prism 9 software (GraphPad Software Inc., San Diego, CA). Data are presented by Mean ± SEM. Kruskal–Wallis with corrected Dunn’s *post hoc* was utilized. *p*-values < 0.05 were considered to be significant.

## Data Availability

The raw data supporting the conclusions of this article will be made available by the authors, without undue reservation.

## Footnotes

1
https://github.com/DavidEilenstein/ImageD

## References

[1] Denney L Ho L-PP. The role of respiratory epithelium in host defence against influenza virus infection. Biom J. (2018) 41:218–33. doi: 10.1016/j.bj.2018.08.004, 30348265 PMC6197993

[2] Galeas-Pena M McLaughlin N Pociask D. The role of the innate immune system on pulmonary infections. Biol Chem. (2019) 400:443–56. doi: 10.1515/hsz-2018-0304, 29604208 PMC9605710

[3] Wright JR. Immunoregulatory functions of surfactant proteins. Nat Rev Immunol. (2005) 5:58–68. doi: 10.1038/nri1528, 15630429

[4] Di Paolo NC Shayakhmetov DM. Interleukin 1α and the inflammatory process. Nat Immunol. (2016) 17:906–13. doi: 10.1038/NI.3503, 27434011 PMC5152572

[5] Werner E Wang H Doetsch PW. Role of pro-inflammatory cytokines in radiation-induced genomic instability in human bronchial epithelial cells. Radiat Res. (2015) 184:621–9. doi: 10.1667/RR14045.1, 26579942

[6] Giuranno L Ient J De Ruysscher D Vooijs MA. Radiation-induced lung injury (RILI). Front Oncol. (2019) 9:877. doi: 10.3389/fonc.2019.00877, 31555602 PMC6743286

[7] Paquet F. Bailey MR Leggett RW Lipsztein J Marsh J Fell TP . 2017) “Annals of the ICRP occupational intakes of radionuclides: part 3,” ICRPPublication137 Ann ICRP2017, pp. 1–486. doi: 10.1177/0146645317734963. 29380630

[8] Radford EP. Potential health effects of indoor radon exposure. Environ Health Perspect. (1985) 62:281–7. doi: 10.1289/ehp.8562281, 4085431 PMC1568705

[9] World Health Organization. WHO Handbook on Indoor Radon: A Public Health Perspective. Geneva, Switzerland: World Health Organization (2009). 23762967

[10] Maier A Wiedemann J Rapp F Papenfuß F Rödel F Hehlgans S . Radon exposure—therapeutic effect and cancer risk. Int J Mol Sci. (2021) 22:1–13. doi: 10.3390/ijms22010316 PMC7796069 33396815

[11] Soussi BG Cordtz RL Kristensen S Bork CS Christensen JH Schmidt EB . Incidence and prevalence of rheumatoid arthritis in Denmark from 1998 to 2018: a nationwide register-based study. Scand J Rheumatol. (2022) 51:481–9. doi: 10.1080/03009742.2021.1957557, 34913402

[12] Maier A van Beek P Hellmund J Durante M Schardt D Kraft G . Experimental setup for radon exposure and first diffusion studies using gamma spectroscopy. Nucl Instrum Methods Phys Res Sect B Beam Interact Mater Atoms. (2015) 362:187–93. doi: 10.1016/j.nimb.2015.09.042

[13] Mirsch J Hintz L Maier A Fournier C Löbrich M. An assessment of radiation doses from radon exposures using a mouse model system. Int J Radiat Oncol Biol Phys. (2020) 108:770–8. doi: 10.1016/J.IJROBP.2020.05.031, 32473181

[14] Papenfuß F Maier A Sternkopf S Fournier C Kraft G Friedrich T. Radon progeny measurements in a ventilated filter system to study respiratory-supported exposure. Sci Rep. (2023) 13:10792. doi: 10.1038/S41598-023-37697-7, 37402813 PMC10319858

[15] Maier A Wiedemann J Adrian JA Dornhecker M Zipf A Kraft-Weyrather W . α-Irradiation setup for primary human cell cultures. Int J Radiat Biol. (2019) 96:206–13. doi: 10.1080/09553002.2020.1683641, 31682776

[16] Rock JR Hogan BLM. Epithelial progenitor cells in lung development, maintenance, repair, and disease. Annu Rev Cell Dev Biol. (2011) 27:493–512. doi: 10.1146/annurev-cellbio-100109-104040, 21639799

[17] Rock JR Onaitis MW Rawlins EL Lu Y Clark CP Xue Y . Basal cells as stem cells of the mouse trachea and human airway epithelium. Proc Natl Acad Sci USA. (2009) 106:12771–5. doi: 10.1073/pnas.0906850106, 19625615 PMC2714281

[18] Nawroth JC van der Does AM Ryan (Firth) A Kanso E. Multiscale mechanics of mucociliary clearance in the lung. Philos Trans R So B Biol Scs. (2020) 375:20190160–8. doi: 10.1098/rstb.2019.0160, 31884926 PMC7017338

[19] Papenfuß F Maier A Fournier C Kraft G Friedrich T. In-vivo dose determination in a human after radon exposure: proof of principle. Radiat Environ Biophys. (2022) 61:279–92. doi: 10.1007/s00411-022-00972-8, 35377069 PMC9021097

[20] Aghapour M Raee P Moghaddam SJ Hiemstra PS Heijink IH. Airway epithelial barrier dysfunction in chronic obstructive pulmonary disease: role of cigarette smoke exposure. Am J Respir Cell Mol Biol. (2018) 58:157–69. doi: 10.1165/rcmb.2017-0200TR, 28933915

[21] Hussong J Lindken R Faulhammer P Noreikat K Sharp KV Kummer W . Cilia-driven particle and fluid transport over mucus-free mice tracheae. J Biomech. (2013) 46:593–8. doi: 10.1016/j.jbiomech.2012.08.020, 23276626

[22] Sears PR Yin W-NN Ostrowski LE. Continuous mucociliary transport by primary human airway epithelial cells *in vitr*o. Am J Physiol Lung Cell Mol Physiol. (2015) 309:L99–L108. doi: 10.1152/ajplung.00024.2015, 25979076 PMC4504973

[23] Adivitiya M Singh K Soura C Shobi V Suneel K. Mucociliary respiratory epithelium integrity in molecular defense and susceptibility to pulmonary viral infections. Biol Basel. (2021) 10:1–37. doi: 10.3390/biology10020095 PMC7911113 33572760

[24] Uzeloto JS Ramos D Silva BSA Lima MBP Silva RN Camillo CA . Mucociliary clearance of different respiratory conditions: a clinical study. Int Arch Otorhinolaryngol. (2021) 25:e35–40. doi: 10.1055/s-0039-3402495, 33542749 PMC7850890

[25] Ball D Mai GT Vinod S Babington S Ruben J Kron T . Stereotactic ablative radiotherapy versus standard radiotherapy in stage 1 non-small-cell lung cancer (TROG 09.02 CHISEL): a phase 3, open-label, randomised controlled trial. Lancet Oncol. (2019) 20:494–503. doi: 10.1016/S1470-2045(18)30896-9, 30770291

[26] Smirnov DA Morley M Shin E Spielman RS Cheung VG. Genetic analysis of radiation-induced changes in human gene expression. Nature. (2009) 459:587–91. doi: 10.1038/nature07940, 19349959 PMC3005325

[27] Reeves SR Barrow KA White MP Rich LM Naushab M Debley JS. Stability of gene expression by primary bronchial epithelial cells over increasing passage number. BMC Pulm Med. (2018) 18:91. doi: 10.1186/s12890-018-0652-2, 29843677 PMC5975426

[28] Barnes PJ. Reactive oxygen species and airway inflammation. Free Radic Biol Med. (1990) 9:235–43. doi: 10.1016/0891-5849(90)90034-G, 2272532

[29] Whitsett JA Alenghat T. Respiratory epithelial cells orchestrate pulmonary innate immunity. Nat Immunol. (2015) 16:27–35. doi: 10.1038/ni.3045, 25521682 PMC4318521

[30] Fernández-Blanco JA Fakih D Arike L Rodríguez-Piñeiro AM Martínez-Abad B Skansebo E . Attached stratified mucus separates bacteria from the epithelial cells in COPD lungs. JCI Insight. (2018) 3:1–18. doi: 10.1172/JCI.INSIGHT.120994, 30185674 PMC6171804

[31] Hu Y-J Wang YD Tan FQ Yang WX. Regulation of paracellular permeability: factors and mechanisms. Mol Biol Rep. (2013) 40:6123–42. doi: 10.1007/s11033-013-2724-y, 24062072

[32] Karakocak BB Keshavan S Gunasingam G Angeloni S Auderset A Petri-Fink A . Rethinking of TEER measurement reporting for epithelial cells grown on permeable inserts. Eur J Pharm Sci. (2023) 188:106511. doi: 10.1016/j.ejps.2023.106511, 37385303

[33] Srinivasan B Kolli AR Esch MB Abaci HE Shuler ML Hickman JJ. TEER measurement techniques for in vitro barrier model systems. SLAS Technol. (2015) 20:107–26. doi: 10.1177/2211068214561025, 25586998 PMC4652793

[34] Pezzulo AA Starner TD Scheetz TE Traver GL Tilley AE Harvey BG . The air-liquid interface and use of primary cell cultures are important to recapitulate the transcriptional profile of *in vivo* airway epithelia. Am J Physiol Lung Cell Mol Physiol. (2011) 300:25–31. doi: 10.1152/ajplung.00256.2010.-Organotypic PMC3023285 20971803

[35] Lee RM Choi H Shin JS Kim K Yoo KH. Distinguishing between apoptosis and necrosis using a capacitance sensor. Biosens Bioelectron. (2009) 24:2586–91. doi: 10.1016/j.bios.2009.01.028, 19233636

[36] O’Sullivan MJ Mitchel JA Das A Koehler S Levine H Bi D . Irradiation induces epithelial cell unjamming. Front Cell Dev Biol. (2020) 8:21. doi: 10.3389/fcell.2020.00021 32117962 PMC7026004

[37] Andarawewa KL Costes SV Fernandez-Garcia I Chou WS Ravani SA Park H . Lack of radiation dose or quality dependence of epithelial-to-mesenchymal transition (EMT) mediated by transforming growth factor β. Int J Radiat Oncol Biol Phys. (2011) 79:1523–31. doi: 10.1016/J.IJROBP.2010.11.058, 21310544

[38] Mitchel JA das A O’Sullivan MJ Stancil IT DeCamp SJ Koehler S . In primary airway epithelial cells, the unjamming transition is distinct from the epithelial-to-mesenchymal transition. Nat Commun. (2020) 11:5053. doi: 10.1038/s41467-020-18841-7, 33028821 PMC7542457

[39] Park J-A Atia L Mitchel JA Fredberg JJ Butler JP. Collective migration and cell jamming in asthma, cancer and development. J Cell Sci. (2016) 129:3375–83. doi: 10.1242/jcs.187922, 27550520 PMC5047682

[40] Boei JJWA Vermeulen S Klein B Hiemstra PS Verhoosel RM Jennen DGJ . Xenobiotic metabolism in differentiated human bronchial epithelial cells. Arch Toxicol. (2017) 91:2093–105. doi: 10.1007/s00204-016-1868-7, 27738743 PMC5399058

[41] Gurushekar PR Isiah R John S Sebastian T Varghese L. Effects of radiotherapy on olfaction and nasal function in head and neck cancer patients. Am J Otolaryngol. (2020) 41:102537. doi: 10.1016/j.amjoto.2020.102537, 32416968

[42] Surico G Muggeo P Mappa L Muggeo V Conti V Lucarelli A . Impairment of nasal mucociliary clearance after radiotherapy for childhood head cancer. Head Neck. (2001) 23:461–6. doi: 10.1002/hed.1060, 11360307

[43] Munkholm M Mortensen J. Mucociliary clearance: pathophysiological aspects. Clin Physiol Funct Imaging. (2014) 34:171–7. doi: 10.1111/cpf.12085, 24119105

[44] Tilley AE Walters MS Shaykhiev R Crystal RG. Cilia dysfunction in lung disease. Annu Rev Physiol. (2015) 77:379–406. doi: 10.1146/annurev-physiol-021014-071931, 25386990 PMC4465242

[45] Passali D de Corso E Platzgummer S Streitberger C Lo Cunsolo S Nappi G . SPA therapy of upper respiratory tract inflammations. Eur Arch Otorrinolaringol. (2013) 270:565–70. doi: 10.1007/s00405-012-2024-5, 22588193

[46] Passali D Gabelli G Passali GC Magnato R Platzgummer S Salerni L . Radioactive Merano SPA treatment for allergic rhinitis therapy. Int J Otolaryngol. (2016) 2016:1–7. doi: 10.1155/2016/2801913, 27698668 PMC5031909

[47] Passali D Gabelli G Passali GC Mösges R Bellussi LM. Radon-enriched hot spring water therapy for upper and lower respiratory tract inflammation. Otolaryngol Pol. (2017) 71:8–13. doi: 10.5604/01.3001.0010.2242, 29116046

[48] Giuranno L Roig EM Wansleeben C Berg A Groot AJ Dubois L . NOTCH inhibition promotes bronchial stem cell renewal and epithelial barrier integrity after irradiation. Stem Cells Transl Med. (2020) 9:799–812. doi: 10.1002/sctm.19-0278, 32297712 PMC7308641

[49] Kiyokawa H Morimoto M. Notch signaling in the mammalian respiratory system, specifically the trachea and lungs, in development, homeostasis, regeneration, and disease. Develop Growth Differ. (2020) 62:67–79. doi: 10.1111/dgd.12628, 31613406 PMC7028093

[50] Eenjes E Tibboel D Wijnen RMH Rottier RJ. Lung epithelium development and airway regeneration. Front Cell Dev Biol. (2022) 10:22457. doi: 10.3389/fcell.2022.1022457, 36299482 PMC9589436

[51] Franken NAP ten Cate R Krawczyk PM Stap J Haveman J Aten J . Comparison of RBE values of high- LET α-particles for the induction of DNA-DSBs, chromosome aberrations and cell reproductive death. Radiat Oncol. (2011) 6:2–6. doi: 10.1186/1748-717X-6-64, 21651780 PMC3127784

[52] Citrin DE Shankavaram U Horton JA Shield W III Zhao S Asano H . Role of type II pneumocyte senescence in radiation-induced lung fibrosis. J Natl Cancer Inst. (2013) 105:1474–84. doi: 10.1093/jnci/djt212, 24052614 PMC3787909

[53] Fouillade C Curras-Alonso S Giuranno L Quelennec E Heinrich S Bonnet-Boissinot S . FLASH irradiation spares lung progenitor cells and limits the incidence of radio-induced senescence. Clin Cancer Res. (2020) 26:1497–506. doi: 10.1158/1078-0432.CCR-19-1440 31796518

[54] Ermund A Meiss LN Rodriguez-Pineiro AM Bähr A Nilsson HE Trillo-Muyo S . The normal trachea is cleaned by MUC5B mucin bundles from the submucosal glands coated with the MUC5AC mucin. Biochem Biophys Res Commun. (2017) 492:331–7. doi: 10.1016/j.bbrc.2017.08.113, 28859985 PMC5596833

[55] Ermund A Trillo-Muyo S Hansson GC. Assembly, release, and transport of airway Mucins in pigs and humans. Ann Am Thorac Soc. (2018) 15:S159–63. doi: 10.1513/AnnalsATS.201804-238AW, 30431338 PMC6855098

[56] Kannapin F Schmitz T Hansmann J Schlegel N Meir M. Measurements of transepithelial electrical resistance (TEER) are affected by junctional length in immature epithelial monolayers. Histochem Cell Biol. (2021) 156:609–16. doi: 10.1007/s00418-021-02026-4 34459960 PMC8695537

[57] Olasagasti F Lieberman KR Benner S Cherf GM Dahl JM Deamer DW . Replication of individual DNA molecules under electronic control using a protein nanopore. Nat Nanotechnol. (2010) 5:798–806. doi: 10.1038/NNANO.2010.177, 20871614 PMC3711841

[58] Frost TS Jiang L Lynch RM Zohar Y. Permeability of epithelial/endothelial barriers in transwells and microfluidic bilayer devices. Micromachines. (2019) 10:1–18. doi: 10.3390/mi10080533, 31412604 PMC6722679

[59] Fung KYY Fairn GD Lee WL. Transcellular vesicular transport in epithelial and endothelial cells: challenges and opportunities. Traffic. (2018) 19:5–18. doi: 10.1111/tra.12533 28985008

[60] Wawrzyniak P Wawrzyniak M Wanke K Sokolowska M Bendelja K Rückert B . Regulation of bronchial epithelial barrier integrity by type 2 cytokines and histone deacetylases in asthmatic patients. J Allergy Clin Immunol. (2017) 139:93–103. doi: 10.1016/j.jaci.2016.03.050, 27312821

[61] Coyne CB Vanhook MK Gambling TM Carson JL Boucher RC Johnson LG. Regulation of airway tight junctions by proinflammatory cytokines. Mol Biol Cell. (2002) 13:3218–34. doi: 10.1091/mbc.e02-03-0134, 12221127 PMC124154

[62] Brune K Frank J Schwingshackl A Finigan J Sidhaye VK. Pulmonary epithelial barrier function: some new players and mechanisms. Am J Phys Lung Cell Mol Phys. (2015) 308:L731–45. doi: 10.1152/ajplung.00309.2014, 25637609 PMC4747906

[63] Palma S Julia W Joana Z Eva T Maike S Paul G . Exposure to carbon ions triggers proinflammatory signals and changes in homeostasis and epidermal tissue organization to a similar extent as photons. Front Oncol. (2016) 5:294. doi: 10.3389/fonc.2015.00294 26779439 PMC4705223

[64] Chauhan V Howland M Wilkins R. A Comparitive Assessement of cytokine expression in human-derived cell lines exposed to alpha particles and X-rays. Sci World J. (2012) 2012:1–9. doi: 10.1100/2012/609295, 22619631 PMC3347887

[65] Boroumand N Baghdissar C Elihn K Lundholm L. Nicotine interacts with DNA lesions induced by alpha radiation which may contribute to erroneous repair in human lung epithelial cells. Ecotoxicol Environ Saf. (2024) 284:117009. doi: 10.1016/J.ECOENV.2024.117009, 39244876

[66] Barthelemy-Brichant N Bosquée L Cataldo D Corhay JL Gustin M Seidel L . Increased IL-6 and TGF-β1 concentrations in bronchoalveolar lavage fluid associated with thoracic radiotherapy. Int J Radiat Oncol. (2004) 58:758–67. doi: 10.1016/S0360-3016(03)01614-6, 14967431

[67] Park J-A Kim JH Bi D Mitchel JA Qazvini NT Tantisira K . Unjamming and cell shape in the asthmatic airway epithelium. Nat Mater. (2015) 14:1040–8. doi: 10.1038/nmat4357, 26237129 PMC4666305

[68] Chandrala LD Afshar-Mohajer N Nishida K Ronzhes Y Sidhaye VK Koehler K . A device for measuring the in-situ response of human bronchial epithelial cells to airborne environmental agents. Sci Rep. (2019) 9:7263. doi: 10.1038/s41598-019-43784-5, 31086226 PMC6513995

[69] Wagner R. Schmedt D. Hanhart P. Walter C. Meisinger C. Bartsch P. (2015). Mitochondrial Protein Import Channels. In: Delcour A. H. (eds) Electrophysiology of Unconventional Channels and Pores. Springer Series in Biophysics, vol 18. Springer, Cham. doi: 10.1007/978-3-319-20149-8_2

