# Supplementary material for: Differentiation and functionality of human bronchial epithelial cells in an air-liquid interface culture are modified by irradiation exposure
Source: Front Public Health. 2026 Jan 12;13:1706687. doi: 10.3389/fpubh.2025.1706687 (PMC12832454; doi:10.3389/fpubh.2025.1706687)
Supplement: Supplementary file 1 [file Data_Sheet_1.pdf]

1 Supplementary Material

Clonogenic survival and determination of isoeffective doses

The colony formation test was used to determine isoeffective doses for α-particles and X-rays. For this purpose, undifferentiated NHBE from two donors were seeded in passage 3 in cell culture vessels at a density of 5×103 cells/ cm2 and irradiated with α-particles (0.25 - 1.00 Gy) and X-rays (0.5 - 4.0 Gy). For details of the experiment protocol, analysis of the colonies and determination of isoeffective doses we refer to previous work (Fournier *et al.*, 2001; Maier *et al.*, 2019). To obtain colonies, the cells were cultivated under standard conditions for 5 days without medium exchange.

As shown in Figure S-1, the isoeffective dose values for a clonogenic survival of 30% are about 0.5 Gy for α-particles and 2.0 Gy for X-rays.

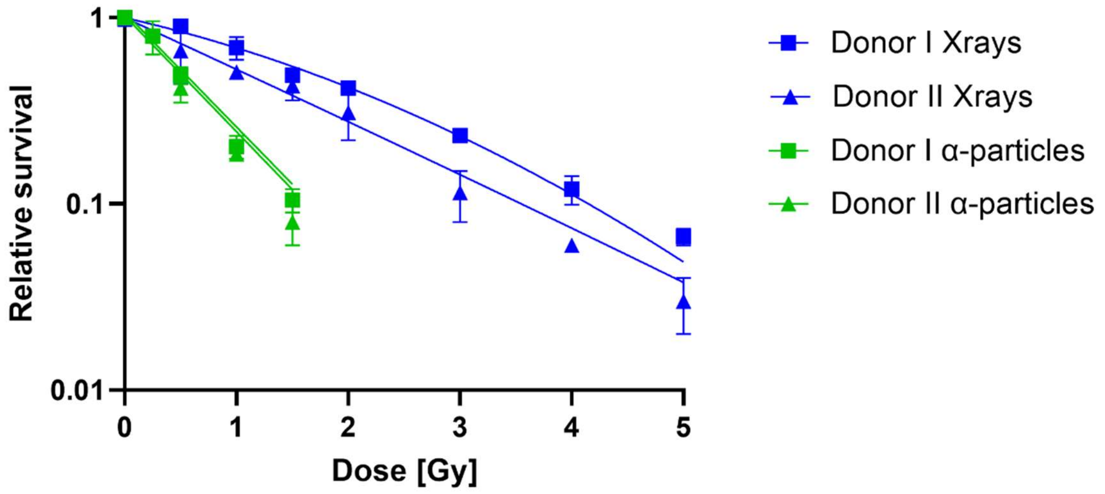

Figure S1: Clonogenic survival of NHBE cells after exposure to X-rays and α-particles. Mean values ± SEM.

Irradiation of basal cells does not lead to a modified release of pro-inflammatory cytokines

Cytokines can modulate the inflammatory response of the epithelium, but also the permeability and the MUC5AC synthesis (Monzon, Forteza and Casalino-Matsuda, 2011; Rincon and Irvin, 2012). A persistent dysregulation of the immune response is associated with the pathogenesis of chronic inflammatory lung diseases. In this study, we aimed to gain deeper insight into the mechanisms underlying radiation induced changes in permeability, considering in particular the role of inflammation and oxidative stress as known side effects of radiotherapy (De Wolf *et al.*, 2015; O'grady, 2019). Therefore, the release of the pro-inflammatory cytokines IFN γ, IL1β, IL6, MCP-1 and TNFα was measured in the cell culture medium at the bottom side of the airlift culture. Their persisting overexpression could also indicate a senescence associated secretory

phenotype (Hansel, Jendrossek and Klein, 2020). Especially club and goblet cells release cytokines and immunomodulatory proteins under oxidative stress.

IFNγ is known as a macrophage activating factor by priming the transcriptional activation of a subset of pro-inflammatory genes like TNFα and IL6 (Ivashkiv, 2018). TNFα induces the activation of IL1β. IL1β, in turn, triggers the release of IL6. An overexpression of IL1β increases the mucus production by regulating the MUC5AC release. This is dysregulated in many chronic inflammatory lung diseases (Gray *et al.*, 2004). IL6 is immune modulatory and induces the release of MCP1, which recruits several immune cells and modulatory factors. The chemokine MCP1 in particular is associated with a radiation-induced inflammatory response and could lead to an increased TEER and FITC dextran flux at the same time (Maus *et al.*, 2001; Hansel, Jendrossek and Klein, 2020; Hansel *et al.*, 2021).

As IL1β is an inflammatory cytokine that might be induced after radiation exposure, the expression of IL1β mRNA was analyzed. Fig. S2 A and B show that in controls the expression level of IL1β raised slightly after seven days after the airlift and remained at this level over time. Notably, no radiation induced change was observed, with exception of an increase after exposure to 2.0 Gy of X-rays at day 7 after air-lift, which is not significant. To find out if the upregulation occurs earlier and is more prominent on the protein level, the release of proinflammatory cytokines into the medium was analyzed 24 h after radiation exposure, at the time of the airlift (48 h after radiation exposure) and afterwards every seventh day throughout differentiation. Similar to the mRNA expression of IL1β, the release of IL1β (Fig. S2 C and D) in controls showed a slight increase seven days after the airlift, remaining at this level over time. The measured values at the end of the observation period were under the detection limit (dotted gray line). Notably, no radiation induced change and inter-individual differences were observed. The respective values for the release of two other inflammatory cytokines, i.e. TNFα and IFNγ, were below the detection limit.

The release of IL6 a cytokine involved in inflammatory processes, is depicted in Figure S2 E-H, separately for donor 1 and 2. The respective values for donor 1 were below the detection limit at 24 and 48 h (dotted gray line), but raised during the following days, so that the obtained values are reliable. The measured maximum was around seven days after airlift. Notably, no radiation induced change was observed. For donor 2, the values were higher and reached a maximum also around seven days after airlift. After exposure to low doses of α-particles and X-rays, no radiation induced change was observed, while after higher doses at least for donor 2 a trend to an increase was observed.

Fig. S2 I and J show the release of the chemoattractant MCP1 protein, also involved in inflammatory processes increasing progressively over the time period of differentiation, but no radiation induced alterations or inter-individual difference were observed.

In conclusion, no significant radiation-induced change was observed with respect to the expression and release of pro-inflammatory cytokines for the time points tested. In this model system, irradiation did not lead to responses related to inflammation or a senescence-associated secretory phenotype. The almost unchanged release of cytokines after irradiation is consistent with the unchanged MUC5AC production. Therefore, cytokine release is unlikely to contribute to the observed altered paracellular permeability after irradiation.

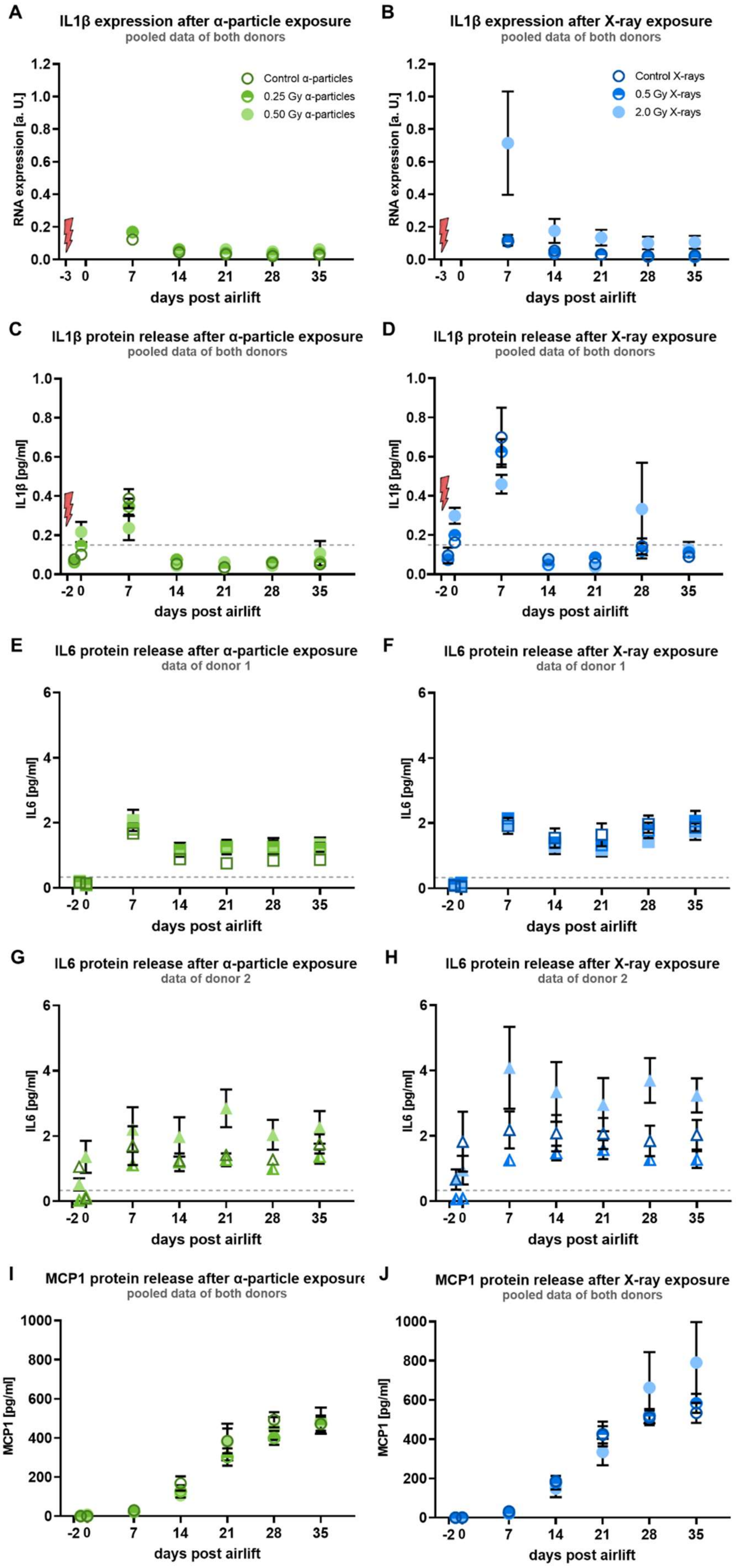
A
IL1β expression after α-particle exposure
pooled data of both donors
RNA expression [a. U.]
Control α-particles
0.25 Gy α-particles
0.50 Gy α-particles
days post airlift
B
IL1β expression after X-ray exposure
pooled data of both donors
Control X-rays
0.5 Gy X-rays
2.0 Gy X-rays
C
IL1β protein release after α-particle exposure
pooled data of both donors
IL1β [pg/ml]
D
IL1β protein release after X-ray exposure
pooled data of both donors
E
IL6 protein release after α-particle exposure
data of donor 1
IL6 [pg/ml]
F
IL6 protein release after X-ray exposure
data of donor 1
G
IL6 protein release after α-particle exposure
data of donor 2
H
IL6 protein release after X-ray exposure
data of donor 2
I
MCP1 protein release after α-particle exposure
pooled data of both donors
MCP1 [pg/ml]
J
MCP1 protein release after X-ray exposure
pooled data of both donors

Figure S2: Radiation exposure did not change significantly the mRNA expression of IL1β nor the release of the pro-inflammatory cytokines IL1β, IL6 and MCP1. (A) and (B) show the mRNA expression of IL1β, which was unaffected by radiation exposure. (C)-(J) show the protein concentration of IL1β, IL6 and MCP1 in the medium, revealing no significant difference between control and irradiated cells. Red flash marks the timepoint of irradiation. The dotted lines show the detection limit of the assay, Data are depicted as mean ± SEM from three independent experiments per donor, resulting in N=6 (except for IL6, where the donors are shown separated N=3) with technical triplicates for the qPCR (n=3) and technical duplicates for the multiplex ELISA (n=2). Kruskal-Wallis with posthoc Dunns test was used for statistical analysis.

References

Fournier, C. *et al.* (2001) 'Changes of fibrosis-related parameters after high- and low-LET irradiation of fibroblasts', *International Journal of Radiation Biology*, 77(6), pp. 713–722. Available at: https://doi.org/10.1080/09553000110045025.

Gray, T. *et al.* (2004) 'Regulation of MUC5AC mucin secretion and airway surface liquid metabolism by IL-1beta in human bronchial epithelia', *American journal of physiology. Lung cellular and molecular physiology*, 286(2). Available at: https://doi.org/10.1152/AJPLUNG.00440.2002.

Hansel, C. *et al.* (2021) 'Metformin Protects against Radiation-Induced Acute Effects by Limiting Senescence of Bronchial-Epithelial Cells', *International journal of molecular sciences*, 22(13). Available at: https://doi.org/10.3390/IJMS22137064.

Hansel, C., Jendrossek, V. and Klein, D. (2020) 'Cellular Senescence in the Lung: The Central Role of Senescent Epithelial Cells', *International journal of molecular sciences*, 21(9). Available at: https://doi.org/10.3390/IJMS21093279.

Ivashkiv, L.B. (2018) 'IFNγ: signalling, epigenetics and roles in immunity, metabolism, disease and cancer immunotherapy', *Nature reviews. Immunology*, 18(9), pp. 545–558. Available at: https://doi.org/10.1038/S41577-018-0029-Z.

Maier, A. *et al.* (2019) 'α-Irradiation setup for primary human cell cultures', *International Journal of Radiation Biology*, pp. 1–8. Available at: https://doi.org/10.1080/09553002.2020.1683641.

Maus, U. *et al.* (2001) 'Alveolar JE/MCP-1 and endotoxin synergize to provoke lung cytokine upregulation, sequential neutrophil and monocyte influx, and vascular leakage in mice', *American journal of respiratory and critical care medicine*, 164(3), pp. 406–411. Available at: https://doi.org/10.1164/AJRCCM.164.3.2009055.

Monzon, M.E., Forteza, R.M. and Casalino-Matsuda, S.M. (2011) 'MCP-1/CCR2B-dependent loop upregulates MUC5AC and MUC5B in human airway epithelium', *American journal of physiology. Lung cellular and molecular physiology*, 300(2). Available at: https://doi.org/10.1152/AJPLUNG.00292.2010.

O'grady, S.M. (2019) 'Oxidative stress, autophagy and airway ion transport', *American journal of physiology. Cell physiology*, 316(1), pp. C16–C32. Available at: https://doi.org/10.1152/AJPCELL.00341.2018.

Rincon, M. and Irvin, C.G. (2012) 'Role of IL-6 in asthma and other inflammatory pulmonary diseases', *International journal of biological sciences*, 8(9), pp. 1281–1290. Available at: https://doi.org/10.7150/IJBS.4874.

De Wolf, K. *et al.* (2015) 'The potential of radiotherapy to enhance the efficacy of renal cell carcinoma therapy', *Oncoimmunology*, 4(10). Available at: https://doi.org/10.1080/2162402X.2015.1042198.
